# Materials Selection for Antifouling Systems in Marine Structures

**DOI:** 10.3390/molecules27113408

**Published:** 2022-05-25

**Authors:** Bradley Donnelly, Karl Sammut, Youhong Tang

**Affiliations:** College of Science and Engineering, Flinders University, Bedford Park, SA 5042, Australia; bradley.donnelly@flinders.edu.au

**Keywords:** antifouling, materials selection, mechanisms, marine sensors

## Abstract

Fouling is the accumulation of unwanted substances, such as proteins, organisms, and inorganic molecules, on marine infrastructure such as pylons, boats, or pipes due to exposure to their environment. As fouling accumulates, it can have many adverse effects, including increasing drag, reducing the maximum speed of a ship and increasing fuel consumption, weakening supports on oil rigs and reducing the functionality of many sensors. In this review, the history and recent progress of techniques and strategies that are employed to inhibit fouling are highlighted, including traditional biocide antifouling systems, biomimicry, micro-texture and natural components systems, superhydrophobic, hydrophilic or amphiphilic systems, hybrid systems and active cleaning systems. This review highlights important considerations, such as accounting for the effects that antifouling strategies have on the sensing mechanism employed by the sensors. Additionally, due to the specialised requirements of many sensors, often a bespoke and tailored solution is preferential to general coatings or paints. A description of how both fouling and antifouling techniques affect maritime sensors, specifically acoustic sensors, is given.

## 1. Introduction

**Figure 1:**
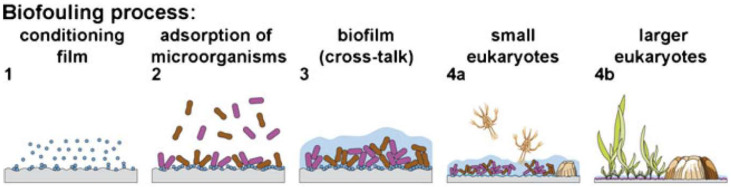
Stages of the typical fouling sequence that occurs on submerged surfaces. Reprinted with permission from Ref. [9]. Copyright 2013 Springer Nature.

**Figure 2:**
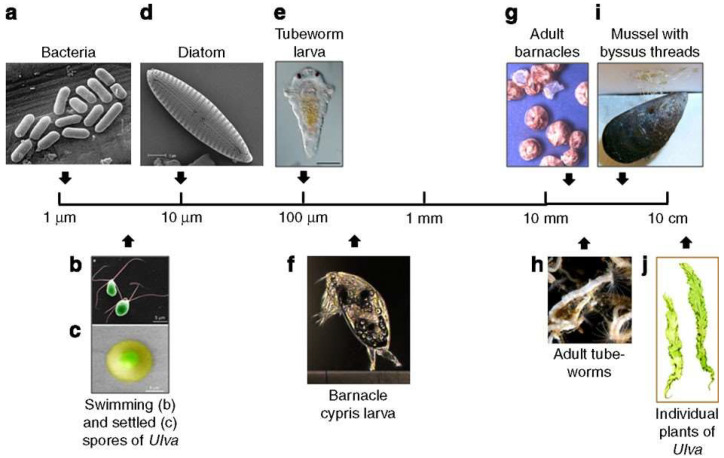
Common marine fouling organisms. Reprinted with permission from Ref. [10]. Copyright 2011 Springer Nature.

Fouling is the accumulation of unwanted substances, such as proteins, organisms, and inorganic molecules, on marine infrastructure such as pylons, boats or pipes due to exposure to their environment [1]. As fouling accumulates, it can have many adverse effects including increasing drag, reducing the maximum speed of a ship and increasing fuel consumption, increasing vulnerability to wave damage, weakening supports on oil rigs and reducing the functionality of many sensors [2]. Fouling can also cause environmental damage, as fouled vessels can act as vectors for introducing invasive species into vulnerable environments [3,4]. The processes that surface and foulants undergo can be generally classified into four “stages” [5,6,7,8], as shown in Figure 1, and fouling information at different stages is shown in Table 1. As soon as a surface becomes submerged, it starts to absorb organic macro-molecules, such as proteins, forming a conditioning layer. This conditioning layer helps the colonisation of micro-organisms onto the surface. The absorption of the organic material and bacteria is highly dependent on the physical features of the surface, the foulant and the conditions. For any submerged surface, there is a region of water that is assumed to have the same velocity as the surface, with a gradient of velocities as the water becomes further from the surface; this region is called the boundary layer. Hydrodynamic interactions between the surface and the water vary the size of the boundary layer at their interface. To reach the surface, bacteria need to cross this boundary layer, which is often a result of van der Waals forces and Brownian motion [5,6]. After some time on the surface, the micro-organisms excrete extra-cellular polymeric substances, mostly polysaccharides, to create a biological matrix that acts as a scaffold for other bacteria to settle on. This scaffold also provides a mechanism where nutrition can be shared between bacteria, creating a stronger and healthier biofilm. The final stage is the settlement of soft and hard macro-fouling eukaryotes [9]. Whilst the fouling process appears to be a linear sequence with one stage dependent on and following the last, it is not strictly sequential [10]. Figure 2 shows common marine fouling organisms. If one of the stages is unable to occur and certain foulants do not adhere, it does not cause the cessation of later stages. For example, various seaweed spores and barnacle larvae are found to settle on a surface at a similar rate and within the same timeframe as various bacteria species [11,12]. Wahl [5] suggested that the rate of colonisation is potentially a reflection of the abundance of the specific foulant in the environment, rather than any sequential process.

## 2. Antifouling

Traditionally, antifouling has been achieved via the use of materials (biocides) that kill organisms that attempt to settle on the surface of a vessel. The most common of these biocides is copper, which has been valued for its antifouling properties for hundreds of years. Copper nails were used by early Roman and Greek shipbuilders, and copper sheathings of a ship’s hull have been used since the 17th century [13,14,15]. Copper is a broad spectrum antifoulant. However, since there are more than an estimated 4000 different fouling species, it is unlikely to be effective against all of them [16,17,18]. In fact, various algae are resistant to copper, and they can cause major failure in the antifouling system; then, if they cover a surface completely and trap the copper underneath, then greater settlement from other foulants is expected. To mitigate this risk, ‘booster biocides’ are often added to target copper resistant organisms. There are many different booster biocides available today, many are herbicidal, targeting the photosynthetic action of algae [19].

### 2.1. Biocide Antifouling Systems

Generally, the biocides are suspended in a substance that either degrades over time and releases the biocide, or the biocide can migrate through the substance to the surface. For a long time, the commercial antifouling market was dominated by tributyl-tin (TBT) self-polishing copolymer coatings (SPC). TBT-SPC paints worked by utilising TBT’s inherently hydrophobic nature to protect the highly water-soluble matrix that it was suspended in. This enabled TBT-SPC paints to remain stable while the active biocide was presented. As TBT leached out of the coating, it would become more vulnerable to fouling and settlement, and it would also become less stable in the water. The depleted portion of coating would quickly hydrolyse and expose more TBT. In addition to enabling the continual release of the biocidal TBT, the surface sloughing would remove any adhered substance as it hydrolysed [13,19,20]. Despite their effectiveness, TBT paints were universally banned due to their significant negative effect on the environment [21]. The paint was toxic and was discovered to have other acute consequences on non-target organisms. Firstly, it was noted that TBT was causing imposex in gastropods, such as whelks. Ideally the TBT would rapidly degrade into less toxic inorganic tin via dealkylation. However, TBT was being absorbed into sediments and the seafloor before this could happen, additionally contaminated organisms would be consumed by larger organisms and TBT would bioaccumulate into the entire food chain [21].

The most common commercial antifouling products are still SPC coatings, but they are now primarily based on copper acrylate. These coatings work in a similar manner to the effective TBT coatings with copper, providing a mediating element for hydrolysis and an acrylate copolymer which rapidly hydrolyses, thereby refreshing the surface. Recently, with the rise of organic copper compounds such as copper pyrithione and the development of copper nanoparticles (NP), the possibilities for the continuously using copper have increased further [22,23]. Copper was able to replace TBT as it provided similar degree of assurance in multiple environments and relied on a similar action for biocide release. Of the possible mechanisms for the antimicrobial action of copper, most relate to it by either interfering with a cell membrane or penetrating it [24,25,26]. These effects may cause cell walls to leak intracellular substances, which, in turn, causes the cell to die. It is also postulated that due to the size of the particles, copper NPs and copper ions can enter a cell either through pores in the cell wall or through ion channels. Once inside the cell, copper interferes with many functions of the cell, such as disrupting DNA, by interfering with mitochondria and creating reactive oxygen species, which further damages cells. NPs also have the potential to interfere with protein folding by altering the metallic ions they use. Additionally, as there are unexpected reactions occurring within the cell, NPs can affect its homeostasis capability, as shown in Acoustic sensors re 3(d) [24,25,26,27]. The antifouling activity of copper can be modulated by changing its form. Chapman et al. [27] studied the differences between nano, micro and macro forms of copper. They found that when suspended in either a sol-gel or polydimethylsiloxane (PDMS), copper NPs’ coatings absorbed the least protein, carbohydrates and slime followed by microparticles then bulk copper.

A significant issue that arises while using copper as an antifoulant is that it can induce galvanic corrosion when applied to aluminium hulled vessels or surfaces. This is worse in marine environments, where the salty water acts as a strong electrolyte. To combat this, copper can be combined with polymer powders and flame sprayed onto a substrate. Wang et al. [28] mixed a polyimide precursor solution with copper powder, and Jia et al. [29] electrolessly coated high-density polyethylene (HDPE) before flame spraying onto steel plates. In both cases, the electrical impedance of the surface increased which in turn increased its resistance to galvanic corrosion. Additionally, both coatings displayed antifouling activity as expected due to the copper component. Elmas et al. spray coated carbon cloth with polyethylene imine (PEI), which passively absorbs copper from the surrounding ocean and is then capable of releasing it when a voltage is applied [30]. They compared various cycle periods and release voltages over a 4-day incubation period. Copper concentration after release was measured in the parts per billion range and the highest release voltage had a 94% reduction in diatom settlement.

After copper, zinc is the next most common metallic biocide in use. Primarily, zinc is employed in the organic form zinc pyrithione (ZnPT), which affects a broad range of organisms, but is prized as a potent algaecide. It functions by disrupting adenosine triphosphate (ATP) synthesis and cell membranes at fairly low concentrations (5–50 µg/L) [31,32]. It is frequently used as a co-biocide with copper as it is effective against some organisms that are resistant to copper.

Silver, particularly silver NPs, is one of the most common metallic biocides that is investigated. It can accumulate in the membranes of microbial cells increasing their permeability, and it also has the potential to completely puncture cell walls thus causing a leaking of intracellular substances resulting in cell death [33]. Silver has low toxicity to eukaryotic cells. This is useful when it is applied in a medical setting as it needs to be safe for the humans it is protecting [34]. It is less effective in the marine environment. However, as some fouling organisms are eukaryotic, it will be resistant.

### 2.2. Biomimicry and Natural Components

Biomimicry is an exploratory or design technique where inspiration is taken from natural organisms that seem to have evolved or developed a solution to a similar problem in a different domain. With regard to antifouling, it was noticed that many sessile organisms are potential sites for the settlement of other fouling organisms and that there is an advantage to organisms that are able to remain free. There are many organisms that remain unfouled despite perpetual exposure to fouling environments. One of the many solutions of organisms employed is the excretion of secondary metabolites which are either toxic to foulants or provide chemical cues to organisms to avoid settling there [1,35].

In an attempt to find natural alternatives to TBT and copper, researchers have started isolating and synthesising the features that these organisms employ to remain fouling free. As of 2014, Qian et al. [36] reviewed over 200 natural marine compounds and 23 synthetic chemicals. It should be noted that a compound derived from natural sources is not automatically conferred environmentally friendly properties as a result of its origin. They may still be highly toxic and can potentially bioaccumulate, especially when used at industrial scales across the whole maritime industry. A follow up [37] to the Qian et al. review examined another 182 natural compounds and highlighted important steps on the pathway to commercialisation: discovery; understanding toxicity, stability and mechanism; and coating incorporation. In order to assess this, the ratio between the concentration of a compound that gives half the maximal desired response (EC_50_) and the concentration of that compound that is lethal to half a population (LC_50_) under the same conditions, EC_50_/LC_50_ is often used as indicator as to whether a compound will be an effective antifoulant and also environmentally suitable. Specifically, an EC_50_/LC_50_ ratio greater than 50 and an EC_50_ amount less than 5 µg/mL is considered a promising natural antifoulant [38]. Secondary metabolites have mostly been isolated from seaweed or macroalgae. Typical natural antifouling compounds classes include steroids, phenolics and furanones [39]. In order to better understand the antifouling action of fouling-resistant organisms, Chapman et al. [40] made synthetic epoxy analogues of the sugar kelp and Guiry’s wrack, Saccharina latissimi and Fucus guiryi. These analogues were then either left blank or doped with a brominated furanone, extracted from Ulva. Rigida. The analogues were verified to be topographically consistent with the algae using scanning electron microscopy (SEM). It was also shown that the analogues that demonstrated the greatest antifouling were the ones that had been doped.

Despite the promising advances that have been made regarding natural compounds for antifouling, there is still significant work that needs to be carried out in order to improve the extraction and manufacturing. Additionally, the natural compounds have not been able to achieve the same level of success seen by the fouling-resistant organisms that they are mimicking [41]. This is largely because fouling-resistant organisms can produce a wide variety of complex compounds, each of which are specifically targeted at a subset of the fouling organisms. Some fouling-resistant organisms are also capable of producing these complex compounds throughout their entire lifespan. Furthermore, as they are living organisms, they will be losing cells and refreshing them, removing any attached material. It would be unreasonable to expect these features to be synthetically replicated in the near future.

Just about every major antifouling manufacturer offers an antifouling paint based on a copper biocide. Azko-Nobel, Hemple, Jotun, PPG and Altrex have available coatings that predominately feature copper as a primary biocide and zinc as a secondary biocide [42,43,44,45,46,47]. This is not an exhaustive list, but it is used to demonstrate that copper and zinc are the industry standard and would be present on the majority of vessels that employ antifouling. To the authors’ knowledge, there are no coatings that employ silver or natural compounds as an antifoulant.

## 3. Delivery Mechanisms and Systems

### 3.1. Degradation of Antifouling Matrix/Coating

**Figure 3:**
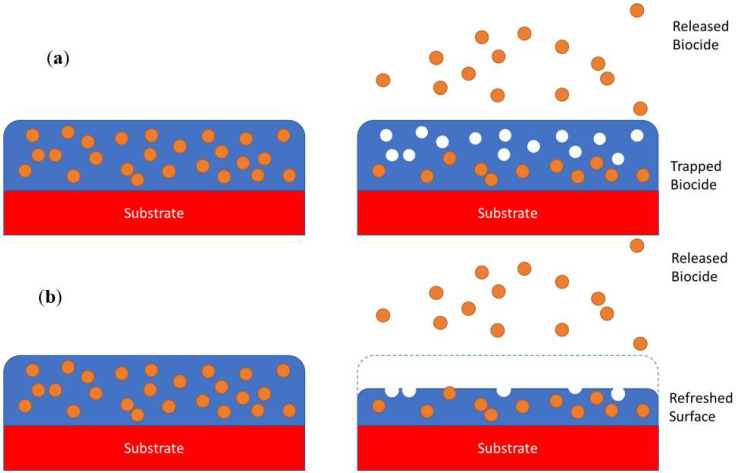
(**a**) An insoluble matrix leaches out biocides from the surface only leaving biocide trapped underneath; (**b**) an erodible coating allows biocides to be continuously released as the coating surface degrades.

Conventional antifouling coatings were comprised of a hard-insoluble matrix containing the biocide, as shown in Figure 3a. At the surface, this biocide would be capable of leaching out and affecting its environment. However, as the surface does not refresh, it quickly depletes the available biocide, leaving the surface inert and with wasted biocide trapped underneath. To remedy this, either the biocide needs to be capable of migrating through the matrix or the matrix needs to gradually degrade to release trapped biocide, as shown in Figure 3b. The latter has been more achievable so far; erodible matrices have been developed to enable coatings to last longer by releasing biocides trapped underneath the inert surface layer. Ashter [48] outlined several degradation modes, as shown in Table 2, that polymers can undergo. Not all of them are applicable to antifouling coatings. To the authors’ knowledge, there are no antifouling systems that make use of either high energy radiation or heat to induce degradation.

**Figure 4:**
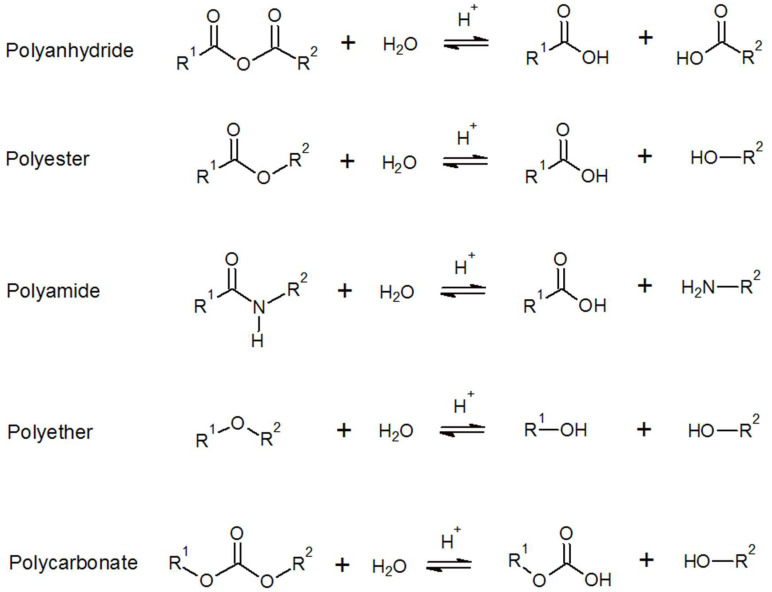
Various hydrolysis reactions [49].

For most polymers, the initial degradation mode they undergo is chemical degradation in the form of hydrolysis. Hydrolysis degrades polymers by breaking the bonds in the functional groups and polymer backbone. This reaction is heavily mediated by other properties of the polymer, particularly hydrophilicity and water absorption. The most sensitive polymers are poly-anhyrdride, -ester, -amide, and -ether. The cleaving reactions are shown in Figure 4.

Biodegradation occurs when enzymes and other organic byproducts, typically from bacteria, depolymerise then mineralise the remaining organic molecules into their inorganic components in addition to carbon dioxide. In the case of marine antifouling systems, mechanical degradation is either caused by shear forces of a vessel moving through the water or the motion of the ocean itself. It often occurs at a macro level and will usually speed up hydrolysis as it exposes more surface to be broken down. Photo-degradation is a less commonly utilised degradation mode for antifouling coatings. It could potentially promote degradation and the release of biocides in areas or conditions that overlap with the optimal growth conditions of many fouling organisms, such as algae. However, as water absorbs significant wavebands of light, there may be inconsistent degradation rates occurring at different depths on a vessel’s hull. Conversely, there may be good reasons for many marine antifouling systems to be resistant to photo-degradation as when a system is most exposed to the most sun, i.e., it is usually out of the water and unable to fully exploit the degradation process because no fouling species are present.

Making polymers that biodegrade adds two antifouling properties. First, the aforementioned re-exposure of trapped biocide or other chemically active antifouling agent that was concealed by the top layers of the polymer. Second, the degradation causes a self-polishing effect that sheds any small foulants that attach before they have a chance to grow. Biodegradation properties can be achieved via the modification of common and stable polymers, extending the effectiveness of other antifouling additives, and increasing the effective lifetime of the system. By reducing the crystallinity of polyurethane (PU) with the addition of Ɛ-caprolactone (CL) and glycolide (GA), the PU degrades more rapidly due to enzymatic reactions and hydrolysis. The nature of these degradations means that the rate of renewal does not depend on vessel motion and can occur on a stationary or docked vessel, unlike many ablative coatings [50]. In an investigation into triblock polyols composition, Yao et al. [51] added 4,5-dichloro-2-octyl-4-isothiazolin-3-one (DCOIT) to polyols containing CL and either polypropylene glycol (PPG) or polyethylene glycol (PEG). Individually, these components are all antifouling in some way; DCOIT is a potent biocide, CL is degradable and can refresh surfaces, and PEG and PPG are hydrophilic. The combination of these factors increases the antifouling activity of the polymer and provides the greater water absorption and degradation rate. By changing the molecular weight of the PEG/PPG segment, this degradation rate can be tuned to provide the correct leaching rate for a given biocide loading. Chen et al. [52] developed a SPC coating that also incorporated a biocide into the co-polymer. They created zinc NPs from zinc and methacrylic monomers. Using these new self-polishing polymers, they incorporated copper oxide or copper pyrthione as the primary biocide. These coatings outperformed commercial copper and copper free coatings in a field test. Interestingly, the zinc only contributed to the polishing effects of the coating and did not provide an additional biocidal agent.

### 3.2. Metal Organic Frameworks

Metal organic frameworks (MOFs) are incredibly promising compounds with potential applications across many domains, such as gas and liquid filtration, drug delivery and encapsulation, supercapacitors and energy storage as well as antifouling. They are a broad family of crystalline compounds made up of metallic ions linked together by organic ligands. They are usually noted for their incredible surface area and porosity but are also finely tuneable, enabling them to be targeted to many different situations.

One of the most promising antifouling features displayed by MOFs is their stimulus response. Specifically, MOFs may have the potential to remain inert until a micro-organism attempts to settle on them, at which point, they become active. This can severely cut down on the amount of biocide they release or even removing the need for release at all by rearranging their surface to dislodge attached material. Sancet et al. [53] employed MOFs anchored to a surface to act as a stimuli responsive biocide releasing agent. MOFs work by using organic linkers to coordinate metal ions into a uniform and repetitive shape. As these shapes are usually porous; Sancet et al. [53] took advantage of this by creating a copper-based MOF that reacts to the biomolecules that foulants release when they attach to a surface. As the MOF reacts, it rearranges its structure and by doing so, it releases Cu^2+^, which is toxic. This ensures that copper is only released when needed and therefore represents an effective method for reducing the amount of copper released into the environment. However, Sancet et al. [53] did not indicate whether this reaction could be tuned to increase efficacy, nor did they gauge the lifetime of the coating. While there is potential that the rearrangement of the MOF structure could provide some antifouling activity by disrupting the settled organisms, it was not clear what, if any, effect this had on the system’s overall antifouling performance. There was also little discussion of the antifouling potential due to the rearrangement of the structure (i.e., self-cleaning).

As with the copper MOFs, the silver MOFs can release silver ions consistently over time [54,55]. Firouzjaei et al. [54] developed a silver NP-based MOF incorporated with graphene oxide. The compound demonstrated 96% antimicrobial action against E. coli; a version of the MOF without graphene also had similar antimicrobial activity. To elucidate the cause of the antifouling effect, Firouzjaei et al. [54] measured the Zeta potential of the compounds. For both compounds, it was found that the Zeta potentials ranged from −40 mV to −70 mV. Zeta-potentials in this range are said to have high colloidal stability, fouling resistance and cell toxicity due to cell membrane disruption [55,56].

Despite the consistent high performing antifouling properties displayed by MOF coatings and MOF-containing polymers, as a viable technology for widespread antifouling use, they are currently unsuitable. While early results are promising, the material is expensive, and many questions remain about its efficacy. As a result, this approach is unlikely to be a cost-effective method that can be applied across the current marine enterprise.

### 3.3. Foul Release/Resistant Systems

Due to genuine environmental concern and the increasing regulations facing biocides, research and development into environmentally safe solutions to antifouling have increased [57,58]. The primary mode of achieving a reduction in the environmental impact of antifouling coatings is to ensure that they leach as little biocide as possible during operation. This minimises the impact that the coating can have on its immediate environment but may still involve some environmentally unfriendly processes in its production. In addition to being more environmentally benign, having a system that is free of a component that is actively being depleted could result in theoretically greater coating lifetimes. The search for more environmentally friendly coatings gave rise to foul release/resistant (FR) coatings. FR coatings work by maintaining specific surface conditions, namely, surface free energy, elastic modulus, roughness and texture, and chemical properties. By carefully selecting these conditions, surfaces are either able to resist the settlement of fouling organisms or reduce the strength of their attachment, so they are simply removed. This removal is often mediated by shear forces presented under regular operating speeds of a vessel [10]. Other possible foul resistance properties come from having surfaces that organisms opt to not settle on. Commercially, there are several available foul release coatings, such as Hempasil, Intersleek and SeaLion, produced by Hempel, International Paint and Jotun, respectively. These coatings typically achieve FR properties by incorporating either polydimethylsiloxane (PDMS) or fluoropolymers into a coating to create surfaces with low surface energy [13,19].

### 3.4. Superhydrophobic

In Baier’s foundational work, he noted that bio-adhesion is the hardest to achieve on surfaces with surface energy between 20 and 30 mNm^−1^, specifically 23 mNm^−1^ [59]. This has been extended to surfaces that are trying to remain clear of fouling, which is a form of bio-adhesion. Surface free energy is typically dependant on the dispersive and polar elements of a material and how they are arranged.

Coatings with surface energies between 20 and 30 mNm^−1^ are typically superhydrophobic, which can promote self-cleaning as well as reducing the strength of adhesion. Regarding antifouling, these are the most common commercially available non-biocidal coatings. Frequently made from fluoro polymers such as polytetrafluoroethylene (PTFE) and poly-2,2,2-trifluoroethyl methacrylate (PFMA) or silicone polymers (mainly polydimethylsiloxane (PDMS)), these coatings form hard smooth surfaces, which limits the adhesion strength of foulants. However, due to manufacturing difficulties and physical vulnerabilities, such as fragility and stability, there have been many recent efforts to improve how they are made and deployed. To simplify the production and use of foul release coatings, as well as to remove the potential for contamination of fluorinated chemicals, Vesco et al. [60] developed and investigated the effect of replacing fluorinated silane with long-alkyl silane chains. The long-alkyl silane coatings were capable of being spray-painted and in certain conditions outperformed the coatings containing fluorinated silanes. The postulated reason for this is that it is due to the increased roughness and the arrangement of the long-alkyl silane chains being more rigid, enabling sheers forces to more effectively remove fouling [58,60].

### 3.5. Hydrophilic

Hydrophobicity, however, is not the only phenomenon responsible for observed fouling resistance. Hydrophilicity can also contribute to antifouling capability by developing a hydration layer that can exert repulsive hydration forces on approaching foulants. The polymer initially employed to achieve this was polyethylene glycol (PEG). PEG forms a hydration layer due to the abundance of polar moieties tightly packed on its surface; these polar moieties strongly attract water molecules. The fouling-resistant effect is a result of preferentially attracting water molecules over potentially fouling molecules. For a fouling organism to attach to a hydrophilic surface, it must displace some of the water in the hydration layer. These coatings have been shown to be very effective at resisting fouling, even under static conditions. However, PEG has yet to demonstrate long term effectiveness in marine environments, it is less chemically stable than most common antifouling coatings and is also quite challenging to apply to a wide variety of surfaces, especially on a large scale. In search of polymers with similar capabilities, superior general performance research has been directed into polyvinylpyrrolidone (PVP), polypeptides and polyacrylics. While some of these polymers have shown equivalent or improved fouling resistance, they are yet to sufficiently tackle the longevity and scale issues.

Another class of surfaces that also rely on hydrophilic interactions to resist fouling are zwitterionic surfaces. Zwitterionic surfaces attract a tight hydration layer via ionic interaction with equal numbers of cationic and anionic groups. These ionic forces are stronger than the typical hydrogen bonding that occurs for other hydrophilic coatings [61,62]. Common zwitterionic groups include x-betaines (sulfobetaines (SBs) and carboxybetaines (CBs)) and phosphorylcholine.

Another feature that contributes to the antifouling mechanism is that zwitterionic charges are internally balanced and are hard to release to facilitate absorption. Additionally, zwitterionic surfaces are more stable in media with high concentrations of salt (NaCl). Whilst they display a greater water-attraction force than traditional hydrophilic surfaces and therefore greater antifouling efficacy, they also remain difficult to apply in large quantities. A key feature that affects the performance of zwitterionic coatings is the distance between the ionic groups on the monomers that make up their bulk. This distance is named the carbon spacer length (CSL). Shao and Jiang [63] investigated the effect of this length on various CB molecules and determined that hydration is affected up to lengths of 3 nm. Recently, Chen et al. [64] developed a simple and fast method for coating steel with polydopamine (PDA) and poly-SB zwitterionic AF polymers. After 30 min of immersion, they were able to create surfaces with lower contact angles and higher bacteria settlement resistance than the bare steel. This shows some impressive improvement to the practicality of employing these advanced coatings. In 2015, Azko-Nobel filled a patent regarding the manufacture of zwitterionic coatings; however, there does not as yet appear to be a commercially available coating that contains zwitterionic functional groups [65].

### 3.6. Amphiphilic

Despite the contrary nature of hydrophobic and hydrophilic surfaces, combining them to create amphiphilic coatings is a common and effective antifouling technique. These coatings exhibit features present in both hydrophobic and hydrophilic coatings, particularly the fouling resistance derived from their hydrophilic component whilst remaining easy to clean, as is the case with hydrophobic surfaces. In addition to this, a fine alternating pattern of interactions on a surface can distort the proteins that various organisms use to adhere to surfaces [58,66,67]. Amphiphilic coatings are commonly constructed by combining PEG with traditional hydrophobic polymers such as PTFE and PDMS. Determining the correct ratio between hydrophilic and hydrophobic components is vital in order to produce the desired effect and to avoid phase separation of the polymer. Guo et al. [67] were able to add PVP, a hydrophilic substance, to a PDMS base, a hydrophobic substance. After the creation of the constituent prepolymers, the final coatings were assembled in a manner approaching what would be expected of a commercial polymer, i.e., mixing with crosslinkers, applying to primed surface, and waiting for it to dry. They observed that the coating with the highest percentage of PVP showed the greatest AF performance. They also showed that the coatings with higher amounts of PVP exhibited phase separation in the form of 2–4 µm cavities forming on the surface, and although these would surely affect the mechanical properties of the coatings, they were not investigated further by the authors. The increased simplicity with which these coatings are applied is a promising direction for advanced coatings.

As zwitterionic coatings are strongly hydrophilic, they have also been incorporated into hydrophobic systems to render them amphiphilic. Koschitzki et al. [68] copolymerised ethylene glycol dicyclopentenyl ether acrylate (DCPEA) with zwitterionic carboxybetaine acrylate (CBA) monomers as well as with both of their corresponding methacrylate monomers (DCPEMA and CBMA). They found that the amphiphilic copolymers of CBMA and DCPEA had the highest AF performance. While all of the tested amphiphilic coatings had superior AF properties compared to a hydrophobic control, there was still a significant difference between the methacrylate and the acrylate groups. A possible explanation given for this difference could have been due to CBMA being more chemically homogenous and smoother than CBA [68]. This study highlights the AF performance of advance amphiphilic polymers as well as the complexity involved in designing them. It is possible that some of the high-end foul release coatings currently on the market are amphiphilic, but without investigating them directly it is hard to determine the functional groups used in non-biocidal coatings as they can be listed as “trade secrets”. Akzonobel market the new Intersleek coatings as amphiphilic, highlighting that they are based on a fluoropolymer, hydrophobic, with added hydrophilic groups which are not specified [69].

### 3.7. Micro-Texture

One of the other key antifouling techniques that has been identified by the previously mentioned paradigm, biomimicry, is microtopography. A feature of many naturally occurring submerged surfaces, microtopography involves intricate ridges and valleys of a specific size, ranging from 1 to 300 µm, that reduce that number of points available for foulants to attach themselves, which is why this is also called the attachment point theory [70,71]. Scardino et al. [70,72] conducted a thorough investigation into the attachment point theory. They tested textures with different surface parameters against the settlement of the most common fouling organisms and showed that certain surface textures were able to inhibit the settlement of some organisms but increased the potential for others. Additionally, it seems to have little to no effect on non-motile organisms. Generally, the width of the texture should be just smaller than the average width of the settling organism. This supports the attachment point theory; if there are fewer spots that organisms are capable to attach, fewer organisms will attach. However, as the action is dependent on the relative size of the organism and the texture features, finding a solution that is effective against a broad array of fouling organisms remains a challenge. As such, a good potential use of microtextured surfaces is to act as a secondary component used alongside a broader antifoulant such as copper oxide.

In addition to the attachment point theory, biomimicry also led to the exploration of the lotus leaf effect. The main feature replicated from the lotus leaves is self-cleaning via droplet roll off. Droplet roll-off is promoted by hierarchical structures that trap tiny air bubbles at the surface, thus imparting the surface with hydrophobic properties. The Sharklet surface, common in medical settings, may be the most recognisable commercial coating utilising this property. However, in general, the effect has not been substantial enough to achieve significant results in maritime conditions. Recent developments in hierarchical surface design, based on Salvinia floating ferns, have enabled coatings to retain a trapped air layer for longer periods when submerged [73]. A new commercial coating making use of these effects, Finsulate, is reporting efficacy up to 5 years. Greco et al. [74] looked at the microtopographical features present on the eye of the crab Carcinus maenas. They measured the surface parameters, roughness, waviness, skewness, aspect ratio, and fractal dimension (based on Scardino). They found that the features presented were similar to microtopographies known to be antifouling; however, they did not carry out any antifouling tests. It would be important to understand how much the microtopographies contribute to the overall antifouling, in combination with mechanical cleaning and chemical processes. Understanding different antifouling solutions developed naturally to combat different fouling species may help hybrid antifouling systems to optimise the most effective combination of technologies. Akuzov et al. [75] sought to improve these coatings by adding non-toxic additives. The additives were chosen for their low toxicity as well as their antioxidant features. It is theorised that antioxidants impair the production of adhesive cements produced by several macro-fouler as well as the extracellular polymer substances (EPS) which are key in biofilm growth. In general, non-biocidal coatings are incredibly promising and may well be the standard that antifouling coatings are measured against in future. However, they are still being outperformed by traditional coatings with respect to price and longevity. Additionally, as these coatings are more complex and are designed to be non-adhesive, they are often challenging to apply and can easily be mis-applied without proper training, causing failure of the coatings shortly after use. Another disadvantage related to the use of these coatings is that they are very sensitive to disruptions to their surface, and while this is true for all coatings, it is especially true for coatings composed of tightly arranged surface ions or hierarchical features.

### 3.8. Hybrid Antifouling Systems

A significant takeaway from many recent reviews is the trend in which different antifouling techniques are applied together in the same environment. There are multiple benefits to doing this; firstly, as different antifouling techniques have different optimal operating conditions, combining them can widen the overall conditions in which a system remains viable. Additionally, as there are growing restrictions regarding the allowable rate copper can leach from antifouling coatings, it has become popular to combine traditional copper biocides with either a booster biocide or a surface effect. By doing this, the copper leaching rate can be limited whilst maintaining similar levels of antifouling capability; while hybrid solutions offer greater protection to marine surfaces, there are often additional costs to consider when combining antifouling techniques. For example, the inclusion of a second component may come at the reduction of the primary antifouling solution which could impact total efficacy. Additionally, as features are added to a coating system, the complexity of synthesising and deploying a system can increase rapidly.

There are several commercially available antifouling coatings that are silicone hydrogel-based coatings that also contain a biocide. While vessels are active, the foul-release components are the dominant feature, and the copper leaching is minor. Conversely, while vessels are stationary, the leaching copper provides protection when the shear forces required for efficient foul-release are not present. A hybrid system based on polyacrylic (PA) polymers combined with poly(N-vinylpyprrolidone) (PVP) and eugenol methacrylate (EM) was developed by Xie et al. [76]. It has hydrophilic surface features to impede fouling attachment as well as eugenol to provide biocidal components. The eugenol was slowly released via hydrolysis largely mediated by the tert-butyldimethylsilyl methacrylate (TBSM). As eugenol has been shown to affect the cell wall of some bacteria, it is possible that it is contact killing on the surface, as well as when released into the water column [77]. As there is a benefit for coatings on vessels to be smooth and low friction, many commercial antifouling products are primarily biocidal with foul release components. Specifically, silylation of the SPC element of AF coatings is common in high-end biocidal paints [78,79,80,81]. Two coatings of note are Hempel’s Hempaguard X7 and International-Marine’s Intercept [82,83]. Hempaguard X7 employs a hydrogel layer on a silicone base, this employs three AF components in a synergistic manner. The hydrogel allows for a slow controlled release of biocide and provides a hydrophilic interface and hydration layer, and the silicone base provides the smooth low energy surface, enabling foul release. Intercept utilises the Lubyon polymer which is marketed as a superhydrophilic polymer, and a silyl-methacrylic copolymer. The Lubyon polymer, in addition to forming a strong hydration layer, also ensures a linear release of biocide, the silyl-methacrylic provides foul release capabilities. The Lubyon polymer is patent protected [82,83], but it is unclear which patent/s provide this protection.

### 3.9. Active Cleaning Systems

For generations, the application of coatings for the management of fouling has been the default solution. In-water hull grooming is the act of regularly cleaning slime and light biofouling from large areas of vessel hulls and represents an alternative to extensive coating applications for vessels. This will often be done in concert with or supplementarily to antifouling coatings and dry dock cleaning. The majority of in-water cleaning is a manual task performed by divers. However, there are several cleaning solutions that greatly reduce the required labour. This includes devices that adhere to vessels hulls and deploy an array of instruments to remove fouling as well as in situ solutions fixed to certain areas of hulls which are capable of cleaning or providing an ongoing inhibitory effect.

The instruments contained on hull-adhered devices include mechanical cleaners, such as rollers and scrapers, as well non-contact instruments, such as cavitating water jets, ultrasonic transducers and lasers [84]. As these devices are often used in conjunction with traditional biofouling techniques and are frequently used dockside, there are two major risks associated with their practice. First, these techniques have the potential to release excess amounts of biocide along with the biofouling, if multiple vessels are all doing this in a contained area, it can quickly become toxic to many organisms. Second, the process of removing biofouling from a surface does not necessarily kill the organisms and their release could pose an invasive species threat to vulnerable ecosystems. For these reasons, many authorities have heavily regulated the process, either requiring it to be carried out outside of an exclusion zone or ensuring that all removed materials are captured in a closed system.

To the authors’ knowledge, all these devices are either teleoperated or manually directed by a diver, none of them are fully autonomous. This is unlikely to be the case for long, as many of the required technologies are already or are approaching appropriate sophistication. There are clearly decent vehicle control models available to support teleoperation, and the artificial intelligence and mission planning capabilities are rapidly progressing. Pivetta et al. [85] recently developed an efficient path planning algorithm for ballast tank inspection, which could very reasonably be applied to this field. These technologies provide a great amount of utility to vessels in extending the lifetime of their other antifouling solutions but are rarely a good choice to be the sole antifouling system employed. However, as the development of cleaning robots progresses, it is possible that compatibility with cleaning robots becomes a significant design consideration for primary antifouling coatings.

The application of in situ cleaning devices is becoming more popular as their implementation and operating costs continue to decrease [84]. The primary antifouling technology used for large hull sections is ultrasound. An in-depth evaluation of active ultrasonic hull cleaning tested the effect of a 20 kHz 16 W transducer producing 200 ms pulses repeated every 2 s. This showed a significant reduction in the amount of fouling present on PMMA and PVC, and while copper plates were also tested, other complications invalidated their results. The authors claimed that the main mechanism responsible is the deterrence or dislodgement of fouling organisms from the surface by the ultrasonic pulses; however, they also suggested the possibility that the antifouling properties could be a result of the ultrasound killing nearby organisms [86]. Interestingly, this raises questions about tuning the features of the pulse if the power is increased; this would probably increase its effectiveness but have a higher running cost. A valuable investigation would be to elucidate the optimal settings regarding power and duty cycle. Guo et al. [87,88] looked into the effect different frequencies and acoustic pressure have on fouling. They also found that a similar frequency (23 kHz) has the greatest inhibitory effect, as compared to 63 and 102 kHz. The reason 23 kHz had better antifouling properties is because it cavitates at lower pressure, so the cavitation causes high shear forces and physically damages fouling organisms. It should be noted that cavitation is also associated with surface erosion; however, the configuration in this case may be different [89]. Park and Lee [90] performed a field trial assessing the effect of ultrasound. They placed 6850 W omnidirectional transducers evenly spaced along the starboard side of a 96,000 m^3^ class drillship. The transducers emitted 23 kHz, 0.5 s pulses with a 50% duty cycle for 4 months. After 4 months of continuously running, the vessel and the transducers along the starboard side had noticeably less fouling compared to the port side. To quantify this, they used a combination of percentage cover and fouling rating, which gives a good representation of how much and what type of fouling was present [91]. The efficacy of acoustic antifouling techniques is, however, highly dependent on the incident frequency as well as energy [92]. Low frequency perturbations, similar to noise generated by engines and motors, actually end up increasing biofouling. This is hypothesised to be due to its similarity to the ambient noise of reefs and other high fouling areas, which may provide settlement cues to organisms [92].

Ultraviolet (UV) radiation also seems to be gaining popularity in niche areas, such as sea chests, anodes, and environmental sensors. UV has been found to be effective at resisting hard biofouling when used intermittently for around 1 min a day. It has also been shown to effectively boost operation of copper coatings and foul release coatings, but prolonged or over-exposure to UV can result in degradation or damage of copper and polymer coatings [93,94]. A significant limiting factor on the uptake of UV is the inconsistency and longevity of the UV light emitting diodes (LEDs) [94]. As the quality of the LEDs improve and optimal activation intervals are determined, UV cleaning systems will become a very promising complimentary system to work alongside traditional AF.

With both ultraviolet and ultrasound cleaning solutions, there must be some additional energy used to power these devices. While this is probably a minor concern, it is rarely considered when considering the overall benefit of the devices. The application of acoustic waves to a surface is known to inhibit biofouling. Table 3 gives a summary of various antifouling systems with the materials selected and their related performance. Technologies are summarised based on three categories: maturity, scale, and efficacy. The maturity category covers the technology readiness level as well as how well the underlying mechanisms are understood. The scale category covers how cost effectively and simply a technology could be applied to a reasonably sized vessel. Efficacy is based on the strength and the longevity of the antifouling effect.

## 4. Marine Sensors

The effect of biofouling on various sensors is multi-faceted. First, fouling occurring directly on sensing elements degrades sensor sensitivity. Slime on an optical sensor/emitter blurs images or attenuates light, while growth on a hydrophone muffles transmission. Second, fouling on or near the sensor can also reduce the accuracy of various sensors. Fouling on the sensor housing can lead to a sensor experiencing an environment unrepresentative of the one which was meant to sense. Some foulants may produce chemicals similar to a specific analyte, and some may shade a sensor altering the lighting conditions.

Recently, Delgado et al. [95] published a thorough review of antifouling strategies for water monitoring sensors. Whilst being focused on sensors used in water for monitoring applications, the review also contains major overlaps with concepts related to sensor fouling protection in general. It highlights important considerations, such as accounting for the effects that antifouling strategies have on sensing mechanism employed by sensors. Additionally, due to the specialised requirements of many sensors, often a bespoke and tailored solution is preferential to general coatings or paints. This is evident as many commercial water monitoring sensors come with antifouling components already incorporated. However, as the focus of that review was on water monitoring applications, it does not cover acoustic sensors in depth. This may be because the main acoustic devices used for water monitoring systems are acoustic Doppler current profilers (ADCP) for flow characterisation and acoustic modems for underwater communication between distributed setups [96]. Compared to other marine sensors, these acoustic sensors may be more tolerant to biofouling interference [95]. Despite this, investigations into the elucidation and quantification of the effects on biofouling on acoustic surfaces are still relevant. Acoustic surfaces, such as submarine sonars, are generally much larger than other sensing devices, which, when fouled, will have a greater effect on the hydrodynamic properties of a vessel. Additionally, as acoustic sensor surfaces are often made of softer elastomers, they are susceptible to damage that may be caused by removing tenaciously adhered fouling organisms. Furthermore, the assumption that acoustic sensors are tolerant to fouling is based on the thickness and density of light fouling and slime, and while these features alone would have a less severe impact, they are not the only concern fouling poses. The effect that fouling can have on an acoustic sensor is the muffling and degradation of the signal reaching the sensor; however, actual investigations into the characterisation of this are rare. To the authors’ knowledge, since 1947, only three publications have been produced that directly discuss this phenomenon. In 1947, Fitzgerald et al. showed that biofouling on a steel plate can absorb 3 dB of a signal after 200 days of immersion. They also showed that antifouling paints worked to slow this degradation [97]. Heupel et al. performed a similar study but with the fouling occurring directly on the sensor housing itself. They found that in addition to fouling reducing signal strength, certain hard foulants also produce acoustic noise, which could further reduce sensor functionality [98]. Deshpande et al. performed a similar study to Fitzgerald, but they assessed the effect of biofouling on neoprene, Perspex and aluminium [99]. Acoustic sensors are sensitive to changes in surface properties and biofouling may affect both the wetting of a surface and may trap air pockets which can hamper transmission. Finally, as the complexity of sonar systems increases, so do the required signal to noise ratios, resulting is greater sensitivity to fouling.

For sensors, the primary goal is to maintain a high signal to noise ratio (SNR) for as long as possible. It is expected that the application of antifouling coatings will negatively impact the initial operation of a sensor, although this is not always the case as in some cases it is possible for antifouling to have no effect on the sensor operation. The benefit is that over longer periods, the treated sensors will degrade slower and be useable for a longer time than that of the untreated sensors. A component that all sensors share is the sensor housing, the structure that surrounds the sensor, providing rigidity and stability. These portions of the sensor are often of least concern for fouling, and rarely come pre-treated by manufacturers. A potential solution to simplify the process of protecting marine sensors housings is to construct them out of materials that have inherently AF properties. Piola et al. [100] utilised 3D printer filaments which contained copper in order to construct plastic components that require no additional antifouling treatment. Testing filaments with different loadings, they found that PLA filaments with 80% copper loading by weight were able to resist heavy fouling for 2 years, while maintaining a copper release rate above 0.02 g/m^2^h for the entire test period. Despite being a promising material for niche marine areas, they did highlight those future investigations into 3D printed AF components should focus on mechanical integrity and longevity as these may be areas of concern.

There seems to be apparent synergy between acoustic sensors—specifically, active acoustic sensors—and ultrasonic cleaning systems. There are anecdotal accounts of deployed maritime acoustic sensors remaining relatively clear of fouling. This is often attributed to either the acoustic energy or the intermittent use of the device. Whilst this is promising, there are certain applications for which it would be unsuitable. Minimising the environmental signature is a requirement for certain vessels. For example, cetaceans are sensitive to acoustic noise and vessels wishing to study them may inadvertently disturb and deter them [101]. Additionally, vessels may operate sonar near the operating frequency of the cleaning ultrasound; it is possible that these systems could interfere with each other. For each of these problems, there may be a solution, if the inhibitory and cleaning effect are still significant with larger intervals between active ultrasonic cleaning. This has yet to be demonstrated in the field. Furthermore, many navies require solutions that limit the detectability of the vessel employing them, in which case, generating consistent ultrasonic noise would be undesirable. One of the difficulties in applying traditional antifouling technologies to acoustic sensors is that many of the commercial paints are not designed to adhere to their elastomeric surfaces. Cold spray embedment is a technology that can apply biocides to surfaces without the need to chemically adhere. This is achieved by accelerating the biocidal particles, usually copper, towards the surface. These particles need to travel above a critical velocity dependent on the surface so that they adequately penetrate the polymer and remain held in place [102]. Vucko et al. applied this technology successfully to seismic polyurethane streams to achieve 210 days of fouling inhibition [103]. However, the focus of this study was on the inhibition of fouling and not the effect that the treatment had on the sensor properties.

Marine optical sensors have a wide variety of uses, such as navigation for robotic systems, visual surveying using a camera or environmental monitoring and contaminant detection [104,105]. In general, the fouling issues that face optical sensors are very similar to the issues that face marine structures, with the added issues that their solutions be transparent, or at least transparent to specific bands of light. Booth et al. [106] performed a study attempting to understand the optical effects of common antifouling biocides Irgarol-1051 and Diuron when incorporated into a transparent polymer optical window. They found that while initially the addition of the biocides decreases the optical transmission, over the course of 6 months of exposure to heavy fouling conditions, they usually achieved better transmission due to being less fouled than the neat polymers. They also indicated that these polymers leach their biocides at a very slow rate and are potentially environmentally safe. While this may be contradictory to other reports on the environmental impacts of Irgarol-1051 and Diuron [107,108], it still highlights a good way of ascertaining whether traditional antifouling techniques can be applied to sensors.

An optical water monitoring sensor contained within a copper housing was developed by Murphy et al. [109]. The sensing components were directly exposed to the environment so their proximity to copper was intended to provide sufficient protection for the bare elements. It should also be noted that the key aspect of this design was not antifouling capability, it was low cost and simplicity, and this may be another reason for the exclusion of antifouling technologies being placed directly on the optical components. Joslin and Polagye [110] developed an optical measurement system that takes advantage of multiple antifouling techniques, including wipers, copper biocide, and the foul release coating ClearSignal. ClearSignal is an optically transparent antifouling coating developed by Severn Marine Technologies. The optical measurement system was deployed for 4 months to gauge the efficacy of the different combinations. Joslin and Polagye encountered issues during this period due to malfunctions in the wiper control system, and while this made it difficult to reach definite conclusions, it did not completely invalidate the experiment. This study showed both the values and flaws of mechanical wipers. Sensor optical ports were clearer when wipers were active. It also indicated that wipers potentially increase the inhibition zone of bare copper, by the distribution of copper particles. However, it also showed that interactions between various antifouling techniques are not always beneficial. After the 4 months, it was found that the wipers were abrading the ClearSignal coating, potentially reducing its efficacy. Additionally, to the authors’ knowledge, this research includes one of the few third-party evaluations of the ClearSignal coating, and while the malfunctions that occurred should not be disregarded, in this instance, ClearSignal coating was outperformed by bare acrylic. It should be noted that a novel metric for determining fouling was employed. This metric directly utilises the optical sensor to determine the amount of fouling present. The downside to this is that the metric is sensitive to anything that reduces the transparency of the optical window. In this case, the abrasion caused by the wipers could be misidentified as fouling. In hindsight, it may have been appropriate to have tested an optical instrument using only ClearSignal and without a wiper to test the efficacy of the coating alone. There is a similar coating that was recently released by PropSpeed, FoulFree is a silicone coating that is specifically designed for acoustic transducers. There is little literature regarding this coating, but it is supported by a third-party transducer manufacturer Airmar [111,112].

Membranes are often a required component of marine sensing equipment. They are used to isolate the required analyte for sensing. As they have relatively large surface areas exposed to marine environments, they are very susceptible to fouling, which can severely hamper their effectiveness. Additionally, as they are often finely tuned to specific operations, they cannot easily be modified to have antifouling properties. However, some success has been found by forming self-assembled monolayers (SAM) of trichloro (1H,1H,2H,2H-perfluorooctyl) silane (TPFS) on commercial dissolved oxygen sensor membranes [113]. It was found that they had a minor effect on the sensing capabilities of the sensor and were able to resist bacterial fouling, which resulted in maintaining sensitivity for a longer period than compared to untreated membranes. Often, the actual sensing device behind the previously mentioned membranes is an electrode. Electrodes are a very broad family of sensors that rely on a large number of interactions. Fundamentally, they are operated by monitoring the electrical properties of a surface and how it changes when in contact with specific particles or conditions. Zhuiykov and Kalantar-Zadeh [114] developed a dissolved oxygen sensor. Starting with an alumina sensing electrode, Cu_2_O and RuO_2_ NPs were deposited on to its surface creating complex nanostructures. It was shown that the sensors with the higher proportion of Cu_2_O had greater fouling resistance and they maintained higher sensitivity than untreated electrodes after exposure.

## 5. Summary and Prospective Work

From this review, it can be seen that research into biofouling and the development of antifouling technologies are both very active fields that can provide a multitude of insights for practitioners. Despite this, there is no magic bullet available when it comes to protecting surfaces from biofouling. Coatings for maritime acoustic sensors are infrequently fully assessed to determine the effect that antifouling treatments have on the sensors. There is a potential mechanism present on many new antifouling surfaces that could dramatically affect the acoustic properties of an acoustic sensor. Complex and sensitive operations are performed on acoustic data and maintaining a high signal to noise ratio is important. Being certain that the antifouling solutions that are applied to sensors do not disrupt their operation is imperative. In order to determine the benefits obtained by including antifouling coating on acoustic sensors, the acoustic properties of their failure states (fouled) need to be determined. It has been some time since the acoustic properties of fouling have been determined. An up-to-date method should be used to include a wider band of frequencies.

If it is found that antifouling coatings have some measurable effect on the acoustic properties of sensors, it is important to determine the dominant properties that lead to that effect and explore whether a system can be designed that minimises it. From the literature, it seems that the simplest approach to ensure antifouling activity would be to take an existing technology and tweak it to alter the acoustic properties in some favourable way. As copper-based coatings are the current market leading solutions and hydrophobic based solutions may have greater impact on acoustic properties, a solution based on copper should be designed. Additionally, since matching acoustic impedance to the signal medium is a key determinate in optimising the acoustic transmission properties of a material, the density, hardness, thickness, and wetting properties of a material should be considered when designing antifouling solutions for acoustic surfaces.

## Figures and Tables

**Table 1:** Fouling information at different stages.

Fouling Phase	Common Foulants	Settling Rate	Dominant Factors	Stage (Refer to Figure 1)
Conditioning Layer	Glycoprotein; Humic, amino and nucleic acids; Polysachides; Lipids	Minutes—hours	Hydrodynamic forces, surface chemical and electrical properties.	1
Biofilm	*Phaeobacter* sp.; *Pseudoalteromonas* sp.; *Nitzschia* sp.; *Amphora* sp.	Hours—days	Hydrodynamic forces, physical surface properties	3
Small Macrofoulers	*Ulva* sp.; *Polysiphonia* sp.; *Ectocarpus* sp.; *Bugula* sp.;	Hours—days	Hydrodynamic forces, physical surface properties, chemical cues	4 a
Large Macrofoulers	*Balanus* sp.; *Mytilus* sp.; *Spirorbis* sp.	Days—weeks	Hydrodynamic forces, physical surface properties, chemical cues	4 b

**Table 2:** Degradation modes of polymeric matrix/coating. Reprinted with permission from Ref. [48]. Copyright 2016 Elsevier.

Mode of Degradation	Factors
Thermal degradation	Exposure to heat
Thermo-oxidative degradation	Exposure to heat and oxygen
Photo-degradation	Exposure to visible light and ultraviolet (UV) light
Irradiation degradation	Exposure of high-energy radiation such as X-rays and gamma irradiation
Mechanochemical degradation	Exposure to mechanical stress
Chemical degradation	Exposure to chemical attack such as solvolysis/hydrolysis, ozonolysis, catalytic degradation
Biodegradation	Exposure to aerobic and anaerobic environment

**Table 3:** Summary of various antifouling strategies with different materials in the systems.

Class	Action	Maturity	Scale	Efficacy
**SPC**	Copper	High	High	High
	Zinc	High	High	High
**CDP**	Copper	High	Med	Med
	Zinc	High	Med	Med
	Organic biocides	Med	Med	Med
**Nanoparticles/composite**	Silver	Med	Low	Med
	Copper	Med	Low	High
	Zinc	Low	Low	Med
	TiO_2_	Med	Low	Med
	Graphene	High	Med	Med
	MOFs	Low	Low	High
**Low surface energy**	Fluorinated polymers (PTFE)	High	Low	High
	Silicone based polymers (PDMS)	High	Med	High
**Surface-topography**	Marine organism inspired (Sharklet)	High	Low	Med
	Lotus leaf inspired (Finsulate)	High	Med	Med
**Hydrophilic**	PEG	High	Low	High
	PVP	Med	Low	High
**Zwitterionic**	Polybetaines	Med	Low	High
	Phosphorylcholine	Med	Low	High
**Active cleaning**	In-water cleaning	High	High	Med
	Ultra-sonic cleaning	High	High	Med
	Ultraviolet cleaning	Med	Low	Med

## Data Availability

The data presented in this study are available on request from the corresponding author.
